# Exploring the Impact of Dance Training on the Structural Plasticity of Empathy‐Related Brain Networks

**DOI:** 10.1155/np/2899142

**Published:** 2026-04-20

**Authors:** Gujing Li, Hui He, Yuanyuan Yu, Yayun Liu, María Luisa Bringas Vega, Benjamin Klugah-Brown, Frank Pollick, Jing Lu, Ling Quan, Lixue Yin, Li Mi, Dezhong Yao, Roberto Rodriguez-Labrad, Mingjun Duan, Cheng Luo

**Affiliations:** ^1^ School of Life Science and Technology, The Clinical Hospital of Chengdu Brain Science Institute, MOE Key Lab for Neuroinformation, University of Electronic Science and Technology of China, Chengdu, China, uestc.edu.cn; ^2^ The Clinical Hospital of Chengdu Brain Science Institute, Department of Psychiatry, Chengdu Mental Health Centre, The Fourth People’s Hospital of Chengdu, Chengdu, China, chengdu.gov.cn; ^3^ Cuban Neuroscience Center, La Habana, Cuba; ^4^ School of Psychology and Neuroscience, University of Glasgow, Glasgow, UK, gla.ac.uk; ^5^ Department of Cardiovascular Ultrasound and Non-Invasive Cardiology, Sichuan Academy of Medical Sciences and Sichuan People’s Hospital, Chengdu, China, samsph.com; ^6^ China-Cuba Belt and Road Joint Laboratory on Neurotechnology and Brain-Apparatus Communication, University of Electronic Science and Technology of China, Chengdu, China, uestc.edu.cn

**Keywords:** dance training, empathy, Granger causality analysis, music training, structural plasticity

## Abstract

Dance integrates bodily movement, rhythmic perception, and emotional expression, engaging complex sensorimotor and affective systems. However, its impact on the structural organization of empathy‐related brain networks remains insufficiently understood. In this study, we adopted a data‐driven approach to construct a structural empathy network based on neuroimaging data from 80 healthy university students and applied Granger causality analysis (GCA) to identify the directional impact of empathy on regional gray matter changes. Empathy ability was assessed using the interpersonal reactivity index (IRI) scale. To further examine training‐induced neuroplasticity, we employed an independent sample consisting of 25 dancers and 25 musicians, alongside 40 matched controls without formal artistic experience. Inter‐regional structural similarity, based on gray matter probability distributions, was calculated to capture modality‐specific plasticity due to dance/music training. Compared to musicians and controls, dancers exhibited significantly higher gray matter probability distributions, which indicates enhanced structural similarity between the left superior temporal gyrus and the left precuneus, left postcentral gyrus, and right paracentral lobule. Notably, this increased structural similarity was also associated with the empathy score (*r* = −0.87, *p* < 0.05, family‐wise error [FWE] corrected). These findings suggest strengthened cross‐modal integration between perceptual and sensorimotor systems, potentially underpinning affective resonance. Our findings highlight the domain‐specific neuroplasticity of the structural empathy network induced by dance training and provide novel insights into how embodied practices shape socio‐emotional brain circuits.

**Chinese Clinical Trials Register:** ChiCTR2200059526

## 1. Introduction

Neuroplasticity refers to the brain’s intrinsic ability to reorganize its structure and function in response to environmental change and underlies the adaptive processes that support learning and expertise. Repeated experience and long‐term training promote both transient functional adaptations and enduring structural modifications, primarily through the formation of new synaptic and neuronal connections [1]. Dance training, as a highly integrative sensorimotor activity, imposes complex demands on the coordination of visual–spatial localization, auditory rhythm processing, and proprioceptive feedback during movement execution. With sustained practice, these multimodal inputs are increasingly integrated into automatized motor patterns [2, 3]. Accordingly, dance provides a powerful natural model for examining experience‐dependent brain plasticity that spans motor, affective, and social domains. Converging neuroimaging evidence suggests that such training drives robust remodeling of the brain’s structural and functional architecture [4, 5].

Dance training elicits robust neuroplastic changes across motor and socioemotional systems, with early adaptations in motor‐related cortices driven by the demands of cross‐modal integration during rhythmic movement [6]. Neuroimaging studies show that dancers demonstrate superior motor performance and increased parietal activation [7], along with enhanced recruitment of the inferior parietal lobule, motor cortex, and cerebellum during observation of familiar movements [8]. Beyond motor expertise, dance critically engages neural systems supporting embodied simulation and empathy, including the mirror neuron system, which has been implicated in affective resonance and social understanding [9]. Structurally, long‐term dance training is associated with regionally specific gray matter reorganization, including reductions in motor regions and increases in affective hubs such as the anterior cingulate cortex and insula. These changes are accompanied by altered functional coupling within interoceptive networks and between the default mode and mirror neuron systems, suggesting training‐related neural efficiency and emotional tuning [10–13]. Complementary diffusion imaging evidence indicates that dance training alters large‐scale white matter pathways, including the superior longitudinal fasciculus and interhemispheric sensorimotor tracts, with patterns that are distinguishable from those associated with music training [3, 14]. However, despite this converging evidence, the organizational principles of these structural adaptations within empathy‐related networks remain unclear, particularly in the context of long‐term dance and music training.

Most prior studies have relied on descriptive or correlational approaches, including voxel‐based morphometry (VBM) and structural covariance analyses, which characterize co‐varying gray matter patterns across regions in development and disease. However, these methods can capture only correlational rather than causal relationships [15, 16]. As a result, it remains unclear how structural changes within empathy‐related regions may hierarchically influence one another, or whether such directional relationships differ between forms of long‐term artistic training. To address this gap, we applied Granger causality analysis (GCA), a method capable of inferring directional influence to delineate directional hierarchies within empathy‐related structural networks. Although originally developed for time‐resolved signals, GCA has been shown to be applicable to structural neuroimaging data when inter‐individual variability is modeled as an ordered series, enabling the inference of directed interregional dependencies [17, 18]. Importantly, this approach has demonstrated robustness and reliability across multiple neurological and psychiatric conditions, including schizophrenia [19] and epilepsy [20]. Building on this established methodological foundation, the present study extends structural GCA to a healthy expert population to examine how long‐term dance and music training differentially shape the architecture of empathic brain circuits.

To further quantify intergroup variation among dancers, musicians, and non‐expert controls, the present study introduces several methodological advancements. First, musician group is included to disentangle the specific contributions of musical elements in dance. Second, by focusing on individuals with long‐term dance training, this study aims to explore structural brain changes that reflect deeper, experience‐dependent neural plasticity. Third, to quantify group differences in structural brain networks among dancers, musicians, and controls, we employ a novel structural similarity analysis based on Kullback–Leibler (KL) divergence [21]. Unlike traditional morphometric analyses that focus on local voxel‐based features, this approach captures inter‐regional structural relationships by comparing the probability density functions (PDFs) of gray matter features. Importantly, this KL‐based structural similarity reflects statistical resemblance in regional gray matter distributions across individuals, rather than structural connectivity or covariance in the sense of fiber pathways or correlated developmental change. As an asymmetric measure of distributional distance, KL divergence enables the assessment of directed structural similarity patterns at the network level [22], providing a complementary perspective on experience‐related brain network architecture.

Taken together, this study integrates GCA and KL‐based structural similarity metrics to examine how dance training alters the structural empathy network. Using T1‐weighted structural magnetic resonance imaging (MRI) data, we first applied GCA to construct directed networks and identified key regulatory hubs based on node‐level in‐degree and out‐degree metrics. We then computed KL divergence matrices to compare morphometric similarity patterns across dancers, musicians, and controls. These findings illuminate both shared and domain‐specific neuroplastic adaptations underlying embodied esthetic training, and offer a novel framework for understanding the intersection of movement, emotion, and social cognition.

## 2. Materials and Methods

### 2.1. Participant Information

In this study, two independent datasets were included. Dataset 1 consisted of structural MRI data from 80 healthy participants and was used to construct structural empathy‐related brain networks. All participants in dataset 1 were students enrolled at the University of Electronic Science and Technology of China (UESTC).

To examine training‐induced neural plasticity, dataset 2 comprised 25 dancers and 25 musicians with long‐term, regular training (mean duration >7 years and average training hours 12 h/week), alongside 40 matched controls without formal artistic training. Participants in dataset 2 were recruited from both UESTC and Southwest Minzu University. The consistency of training history was verified via questionnaires and independent expert evaluations by certified instructors. Dancers completed three domain‐specific assessments: a flexibility task, a structured imitation task, and a spontaneous improvisation task. Musicians completed a continuous performance task and a sight‐reading task.

All participants met the following inclusion criteria: right handedness as assessed by the Edinburgh Handedness Inventory [23]; absence of metallic implants; no contraindications to MRI scanning; no history of brain injury, developmental disorders, neurological or psychiatric conditions; no chronic diseases, such as hypertension or heart disease; and no history of alcohol or drug abuse.

The protocol was reviewed and approved by the UESTC Ethics Committee. Consistent with our previous studies [24], all participants provided written informed consent in accordance with the Declaration of Helsinki (World Medical [25]).

### 2.2. Interpersonal Reactivity Index (IRI) Scale

Participants’ empathic traits were assessed using the Chinese IRI (C‐IRI), a widely used self‐report measure originally developed by Davis [26, 27]. The C‐IRI comprises 28 items rated on a five‐point Likert scale (0 = completely inconsistent, 4 = completely consistent), yielding scores across four subscales: perspective taking (PT), fantasy (FS), empathic concern (EC), and personal distress (PD). PT and FS reflect cognitive empathy, the capacity to adopt others’ viewpoints and engage with imagined scenarios; while EC and PD represent affective empathy, distinguishing between other‐oriented concern and self‐oriented distress responses.

### 2.3. Imaging Collection and Preprocessing

High‐resolution T1‐weighted anatomical images were acquired using a three‐dimensional fast spoiled gradient echo (3D FSPGR) sequence. For the cohort of 80 healthy participants (dataset 1), a total of 156 slices were obtained, while the dancer, musician, and control subgroups were scanned with 152 slices (dataset 2). The remaining scanning parameters were as follows: repetition time (TR) = 6.008 ms, echo time (TE) = 1.984 ms, flip angle (FA) = 9^°^, field of view (FOV) = 256 × 256 mm^2^, matrix size = 256 × 256, and slice thickness = 1 mm with no interslice gap.

Structural image preprocessing was conducted using the Statistical Parametric Mapping software (SPM12) toolbox [28] (http://www.fil.ion.ucl.ac.uk/spm/software/spm12). The preprocessing pipeline consisted of the following steps: (1) visual inspection for quality control, with exclusion of images exhibiting artifacts; (2) conversion of raw T1‐weighted data from DICOM to NIFTI format, generating co‐prefixed images for downstream analysis; and (3) anterior commissure–posterior commissure (AC–PC) alignment, in which the image origin was manually adjusted to the AC to ensure anatomical normalization across subjects.

### 2.4. VBM Analysis

**Figure 1:**
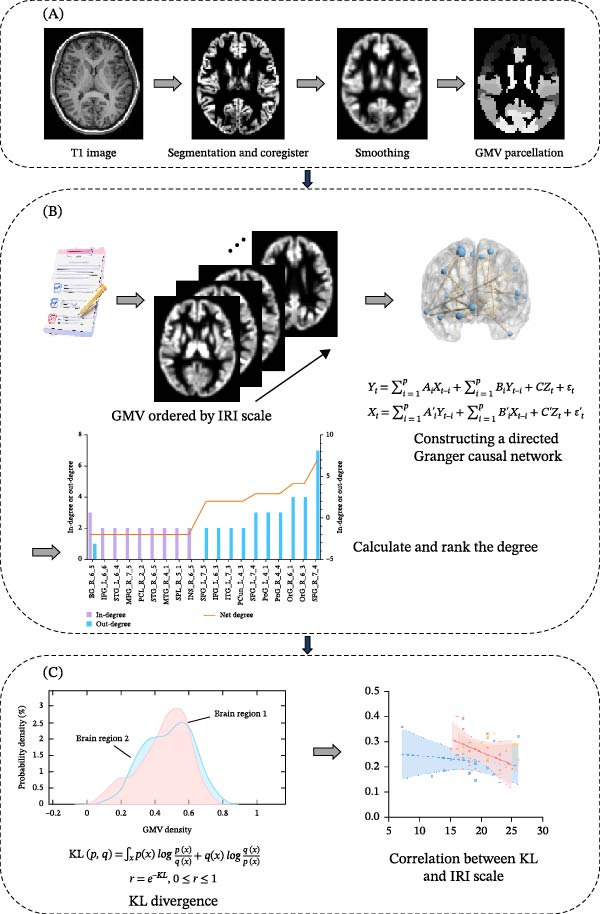
Analysis procedures. (A) Voxel‐based morphometry was used to estimate regional gray matter volume. (B) Granger causality analysis was conducted to construct a causally informed structural empathy network. (C) Structural similarity between brain regions was computed, followed by correlation analyses between structural similarity measures and empathy scale scores.

VBM is a widely applied automated technique for the analysis of structural MRI data, enabling the quantitative assessment of local tissue composition or volume in specific brain regions [29]. The core principle of VBM involves transforming individual brain images into a standard anatomical space through spatial normalization, tissue segmentation, and smoothing, thereby allowing voxel‐wise comparisons of gray matter volume across individuals. As illustrated in Figure 1A, this study employed the computational anatomy toolbox (CAT12) embedded within the SPM12 software package (http://dbm.neuro.uni-jena.de/cat12/) to perform gray and white matter segmentation and to calculate regional gray matter volume. The detailed preprocessing pipeline included the following steps: (1) tissue segmentation: T1‐weighted images were segmented into gray matter, white matter, and cerebrospinal fluid; (2) spatial normalization: the original and segmented images were spatially registered to a standardized brain template, with voxel sizes resampled to 1.5 mm^3^; (3) modulation: to correct for volumetric distortions introduced during normalization, the gray matter images were modulated to preserve total tissue volume; (4) sample quality check: as this study focused exclusively on gray matter, only the quality of segmented gray matter images was evaluated; (5) estimation of total intracranial volume (TIV): gray matter, white matter, and cerebrospinal fluid volumes were used to estimate whole‐brain intracranial volume, which was included as a covariate in subsequent analyses; and (6) Spatial smoothing: an 8 mm full‐width at half‐maximum (FWHM) Gaussian kernel was applied to the modulated gray matter images for spatial smoothing.

To perform regional gray matter volume estimation, this study adopted the Brainnetome Atlas (BN) [30], which parcellates the brain into 246 distinct anatomical regions. Among these, 210 cortical regions were assigned to one of seven canonical functional networks defined by Yeo et al. [31]: the visual network (VN), sensorimotor network (SMN), dorsal attention network (DAN), ventral attention network (VAN), limbic network (Limbic), frontoparietal network (FPN), and default mode network (DMN). The remaining 36 regions, located subcortically, were collectively classified in this study as the subcortex network (SCN).

### 2.5. GCA

According to Figure 1B, GCA [18] was applied to the dataset of 80 healthy participants to construct a structural core network for empathy. The approach assesses directional influences between morphometric sequences ordered according to empathy scores. GCA is a multivariate linear regression‐based method that estimates directed connections between brain regions by evaluating the influence of past values of one time series on another. The central premise of GCA is that if the inclusion of past values from one time series significantly improves the autoregressive prediction of another, a directed predictive influence is inferred. This technique has been widely employed in functional brain network analyses using fMRI [19, 32–34].

The method is mathematically formalized in Equations (1) and (2), where, *Y*_*t*_ and  *X*_*t*_ represent the values of two time series at time point *t*, and *A*_*i*_ and $\;A_{i}^{\prime }$ are the path coefficients in the regression model used to estimate the causal effect. The terms *B*_*i*_ and $B_{t}^{\prime }$ are autoregressive coefficients, *Z*_*t*_ denotes covariates at time point *t*, and *ε*_*t*_ is the residual term. The parameter *p* indicates the order of the autoregressive model, that is, the number of lagged time points considered (in this study, *p* = 1 was set as a fixed value).

(1) Yt= ∑i=1pAiXt−i+∑i=1pBiYt−i+CZt+εt,

(2)

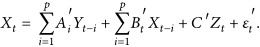




Participants were first sorted according to ascending empathy questionnaire scores. For each brain region, the corresponding gray matter volumes across participants were then arranged into a sequence. The resulting sequence of regional gray matter volumes thus representing a monotonic ordering of empathy scores across participants, capturing its progressive structural modulation across the brain. Accordingly, the *Y*_*t*_ and *X*_*t*_ time series in this analysis represented empathy‐ordered gray matter volume sequences, while the covariates *Z*_*t*_ included age, sex, total TIV, and the interval between adjacent sequence elements (i.e., the average empathy score difference).

A significance threshold of *p* < 0.001 was applied to construct directed connections between regions of interest (ROIs) and to calculate network node metrics. Specifically, in‐degree refers to the number of edges directed toward a given node, out‐degree to the number of edges projecting from the node to others, and net degree is defined as the difference between out‐degree and in‐degree. A negative net degree indicates that a region acts as a source of direct influence, while a positive value identifies it as a target of causality.

Regions with the absolute value of net degree greater than or equal to 2 were identified as empathy‐relevant brain regions, as illustrated in Figure 1, with the net degree values presented in absolute terms to highlight nodes with prominent directional influence.

### 2.6. Structural Similarity Analysis

To investigate whether dance training induces plasticity in the structural empathy network, this study employed a structural similarity analysis framework proposed by Kong et al. [21], applied to the dataset of dancers, musicians, and control participants. A total of 19 ROIs identified via GCA were used for cross‐group comparison. The core principle of this framework is to estimate the PDF of local morphometric features within each brain region and to quantify inter‐regional relationships via KL divergence, as illustrated in Figure 1C. The analysis was conducted in three main steps:

Step 1: morphometric computation: using VBM, gray matter segmentation was performed, and voxel‐wise gray matter intensity values within each ROI were extracted.
Step 2: PDF estimation: for each ROI, the probability density distribution of voxel intensities was estimated using a kernel‐based density estimation method (Ksdensity). The kernel density estimation is formalized in Equation (3), where *x*_*i*_ represents the gray matter intensity of voxels, *n* denotes the number of voxels within the ROI, *K* is the kernel smoothing function, and *h* is the bandwidth parameter.

(3) f∧x= 1nh∑i=1nKx−xih.

Step 3: quantifying structural similarity between brain regions: the KL divergence was used to quantify the difference between gray matter distributions of any two ROIs. The exponential of the KL divergence was adopted as a measure of structural similarity between the two regions. This procedure is expressed in Equations (4) and (5), where  *p*(*x*) and *q*(*x*) denote the PDFs of two different ROIs, and *r* represents the final structural similarity index.

A higher KL value indicates greater structural similarity between two regions, whereas lower values suggest morphometric divergence, potentially reflecting training‐related structural reorganization.

(4) KLp,q= ∫Xpxlogpxqx+qxlogqxpx,

(5) r= e-KL, 01.≤r≤

### 2.7. Statistical and Correlation Analyses

Statistical analysis of the structural similarity matrices across dancers, musicians, and control participants was first conducted using one‐way analysis of variance (ANOVA), with age, sex, years of education, and TIV included as covariates. Given that the structural similarity indices deviated from normality and that group sizes were unequal, statistical significance was assessed using a nonparametric permutation framework with 10,000 iterations. Specifically, empirical *F*‐values were compared against null distributions generated by randomly permuting group labels to obtain permutation‐based *p*‐values. The significance threshold was set at *p* < 0.05, and post hoc comparisons were conducted for effects that survived permutation testing. Detailed permutation results, including the rank of the observed *F*‐values within the null distributions, are reported in Supporting Information. Brain network visualization was performed using BrainNet Viewer version 1.7 [35], a tool designed for the visualization of brain networks.

Next, partial correlation analyses were used to examine the association between structural similarity and C‐IRI scores, while controlling for the same four covariates: age, sex, years of education, and TIV. Statistical significance was assessed using family‐wise error (FWE) correction, with a corrected threshold of *p* < 0.05.

## 3. Results

### 3.1. Demographic Information and Scale Scores

As shown in Table 1, four subjects from the initial dataset of 80 healthy participants were excluded due to excessive head motion (framewise displacement >1.5 mm) during MRI scanning, resulting in a final dataset comprising 76 individuals. In the dataset 2, including dancers, musicians, and control subjects, one participant from the control group and one from the musician group were excluded based on head motion criteria. Thus, the final analysis included 25 dancers, 24 musicians, and 39 control subjects. As depicted in Table 2, there were no significant differences among the three groups regarding age, gender, years of education, and mean framewise displacement. Tables 3 and 4 demonstrated the IRI scale scores of dataset 1 and dataset 2. We also report the effect size of partial eta square of IRI scales in Table 4.

**Table 1:** Demographic information and behavioral data of dataset 1.

Demographic variables	Healthy participants
Gender (male/female)	37/39
Age	23.34 ± 1.10
Education years	17.38 ± 1.11
Mean FD	0.047 ± 0.021

*Note*: The values shown in the table are the mean ± standard deviation.

**Table 2:** Demographic information and behavioral data of dataset 2.

Demographic variables	Dancer group	Musician group	Control group	*p*‐Value
Gender (male/female)	8/17	15/9	19/20	0.182^a^
Age	20.04 ± 2.49	19.92 ± 1.44	20.51 ± 1.55	0.396^b^
Education years	13.76 ± 2.33	13.83 ± 1.63	14.69 ± 1.44	0.069^b^
Mean FD	0.042 ± 0.015	0.051 ± 0.021	0.043 ± 0.020	0.158^b^

*Note*: The values shown in the table are mean ± standard deviation.

^a^Statistical tests using chi‐square test.

^b^Statistical tests using one‐way ANOVA.

**Table 3:** Behavioral data of dataset 1.

C‐IRI scale	Scores
Perspective taking (PT)	20.22 ± 2.94
Fantasy (FS)	17.55 ± 3.61
Empathic concern (EC)	19.96 ± 3.28
Personal distress (PD)	15.33 ± 3.65
Total score	81.44 ± 8.16

*Note*: The values shown in the table are the mean ± standard deviation.

**Table 4:** Behavioral data of dataset 2.

C‐IRI scale	Dancer group	Musician group	Control group	*p*‐Value	$\eta_{\text{partial}}^{2}$
Perspective taking (PT)	18.75 ± 2.89^b^	17.93 ± 4.77^c^	20.55 ± 2.93^d^	0.091^a^	0.10
Fantasy (FS)	15.06 ± 3.49^b^	17.13 ± 4.41^c^	17.90 ± 4.76^d^	0.145^a^	0.08
Empathic concern (EC)	19.00 ± 3.61^b^	20.73 ± 4.54^c^	20.45 ± 3.55^d^	0.405^a^	0.04
Personal distress (PD)	14.00 ± 2.56^b^	15.00 ± 4.36^c^	15.65 ± 3.70^d^	0.400^a^	0.04
Total score	66.81 ± 6.70^b^	70.80 ± 10.65^c^	74.55 ± 10.81^d^	0.067^a^	0.11

*Note*: The values shown in the table are mean ± standard deviation.

^a^Statistical tests using one‐way ANOVA.

^b^Scale data for 16 subjects.

^c^Scale data for 15 subjects.

^d^Scale data for 20 subjects.

### 3.2. Core Brain Regions of the Structural Empathy Network

**Figure 2:**
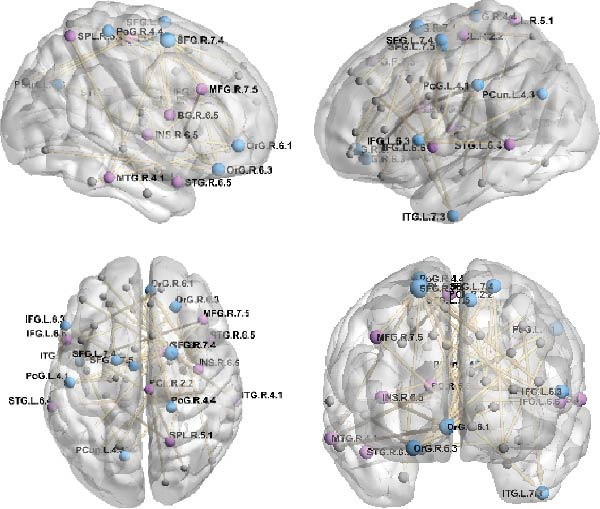
Directed connected networks obtained by Granger causal analysis (blue colors indicate nodes with positive access, and purple colors indicate nodes with negative access; the larger the node, the larger the absolute value of its access degree).

Using GCA, 19 brain regions with gray matter volumes were identified as being significantly associated with empathy scores in healthy participants. Figure 2 presents the whole‐brain directional connections maps, with arrowheads indicating GCA‐derived direction of influences. Several regions emerged as structurally influential regions, including the right dorsal caudate nucleus, left inferior frontal gyrus (IFG), bilateral superior temporal gyri (STG), right middle frontal gyrus, and right paracentral lobule. The gray matter volume patterns of these regions significantly influence structural changes in other regions associated with empathy. In contrast, target regions within the directed structural network such as the bilateral superior frontal gyri (SFG), right orbital gyrus, bilateral postcentral gyri, and left precuneus showed gray matter trajectories shaped by structural covariation with other brain areas.

**Figure 3:**
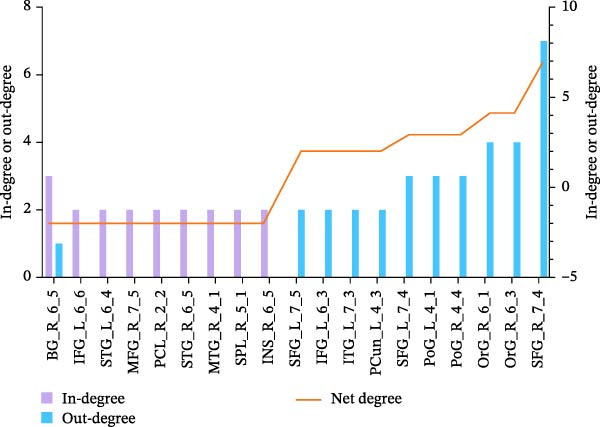
Brain regions of in‐degree, out‐degree, and net degree by Granger causal analysis.

As shown in Figure 3, the in‐degree and out‐degree of each significant directed edge were computed, and brain regions were ranked in ascending order based on their net degree values. Regions with larger absolute net degree values were designated as key nodes in the structural empathy network. These included the bilateral SFG, right orbital gyrus, bilateral postcentral gyri, left precuneus, left inferior temporal gyrus, left IFG, right dorsal caudate nucleus, bilateral STG, right middle frontal gyrus, right paracentral lobule, right middle temporal gyrus (MTG), right superior parietal lobule, and right insula. Full details are provided in Table 5.

**Table 5:** Brain regions of in‐degree, out‐degree, and net degree by Granger causal analysis.

Brain regions	Abbreviations	In‐degree	Out‐degree	Net degree
Basal ganglia	BG_R_6_5	1	3	−2
Inferior frontal gyrus	IFG_L_6_6	0	2	−2
Superior temporal gyrus	STG_L_6_4	0	2	−2
Middle frontal gyrus	MFG_R_7_5	0	2	−2
Paracentral lobule	PCL_R_2_2	0	2	−2
Superior temporal gyrus	STG_R_6_5	0	2	−2
Middle temporal gyrus	MTG_R_4_1	0	2	−2
Superior parietal lobule	SPL_R_5_1	0	2	−2
Insular gyrus	INS_R_6_5	0	2	−2
Superior frontal gyrus	SFG_L_7_5	2	0	2
Inferior frontal gyrus	IFG_L_6_3	2	0	2
Inferior temporal gyrus	ITG_L_7_3	2	0	2
Precuneus	PCun_L_4_3	2	0	2
Superior frontal gyrus	SFG_L_7_4	3	0	3
Postcentral gyrus	PoG_L_4_1	3	0	3
Postcentral gyrus	PoG_R_4_4	3	0	3
Orbital gyrus	OrG_R_6_1	4	0	4
Orbital gyrus	OrG_R_6_3	4	0	4
Superior frontal gyrus	SFG_R_7_4	7	0	7

### 3.3. Structural Similarity Comparisons Between Empathy Core Regions

**Figure 4:**
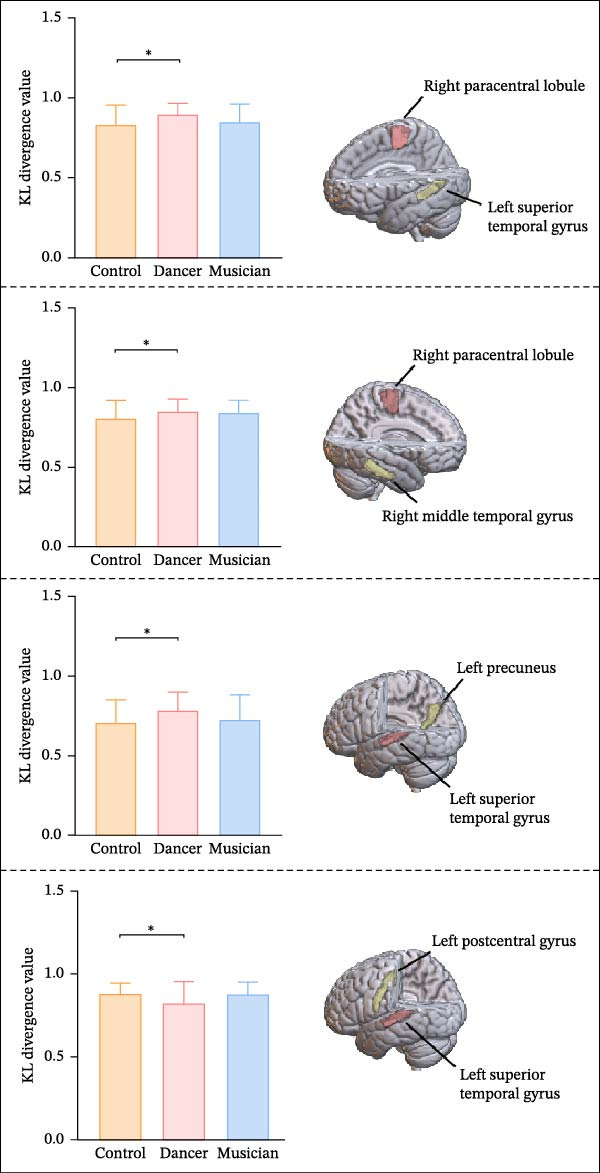
ANOVA and post hoc test results for structural similarity between brain regions localized by Granger causal analysis ( ^∗^ *p* < 0.05).

As shown in Figure 4, significant group differences were found in structural similarity between the right paracentral lobule and left superior temporal gyrus, the right paracentral lobule and right MTG, the left superior temporal gyrus and left precuneus, and the left superior temporal gyrus and left postcentral gyrus.

Post hoc analyses further revealed that, compared to the control group, the dancer group exhibited increased structural similarity between the right paracentral lobule and left superior temporal gyrus, between the right paracentral lobule and right MTG, and between the left superior temporal gyrus and left precuneus. In contrast, structural similarity between the left superior temporal gyrus and left postcentral gyrus was significantly reduced in the dancer group.

### 3.4. Correlation Between Structural Similarity and Empathy Scores

**Figure 5:**
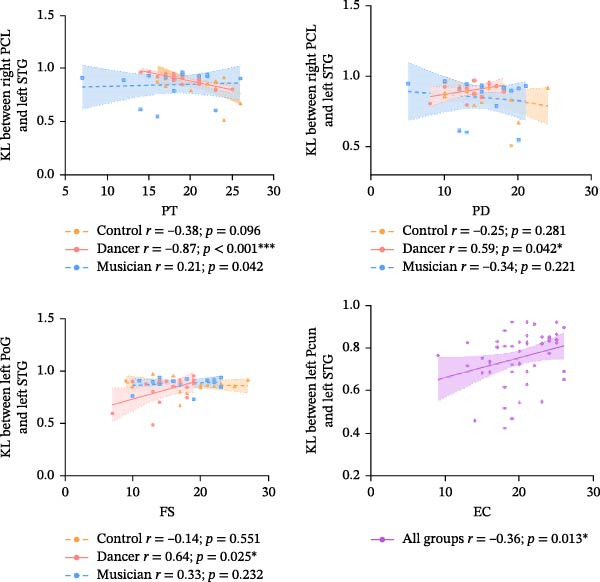
Correlation results between structural similarity and empathy scale scores ( ^∗^ *p* < 0.05,  ^∗∗∗^ *p* < 0.001). Solid lines indicate that the dynamic functional connectivity of the group was significantly correlated with the empathy scores, and dashed lines indicate nonsignificant correlations. EC, empathic concern; FS, fantasy; PCun, precuneus; PD, personal distress; PCL, paracentral lobule; PoG, postcentral gyrus; PT, painstaking; STG, superior temporal gyrus.

As illustrated in Figure 5, correlation analyses between structural similarity and C‐IRI subscale scores revealed the following patterns within the dancer group showed that only the negative association between structural similarity of the right paracentral lobule and the left superior temporal gyrus and PT scores survived FWE correction (*r* = −0.87, *p* < 0.01, FWE‐corrected).

Other results, including the positive correlation between structural similarity of the right paracentral lobule and the left superior temporal gyrus and PD scores, the positive correlation between the left superior temporal gyrus and the left postcentral gyrus and FS scale scores, as well as the positive correlation between the left superior temporal gyrus and the left precuneus and EC scores across all participants, were significant only at the uncorrected threshold (*p* < 0.05) and are, therefore, reported as exploratory findings.

## 4. Discussion

Using T1‐weighted structural imaging data, this study applied GCA to construct a directed brain structural network, revealing progressive morphology‐based influences of empathic traits in healthy individuals. Within this network, 19 ROIs were identified based on in‐degree and out‐degree values, highlighting their structural associations with empathy. These ROIs encompassed both structurally influential regions, such as the right dorsal caudate nucleus, left IFG, and bilateral STG, and target regions within the directed structural network, including the bilateral SFG, right orbital gyrus, and bilateral postcentral gyri.

Beyond identifying the core network, we examined the plastic effects of dance training by comparing interregional structural similarity across dancers, musicians, and matched healthy controls. The results demonstrated that dance training selectively enhanced intra‐hemispheric gray matter similarity, with distinct association with emotional and cognitive components of empathy.

### 4.1. Localization of the Structural Empathy Network via GCA

GCA revealed that several regions acted as structurally influential regions within the empathy network. Notably, the right dorsal caudate, left IFG, and bilateral STG exhibited gray matter volume changes that showed significant directed associations with downstream regions in the structural network.

The caudate nucleus, a central component of the basal ganglia, is involved in fine motor coordination, reward processing, and learning [36]. Its gray matter volume has been linked to prosocial behavior in trust‐based decision‐making [37], and to vicarious responses to social exclusion [38], suggesting its relevance for affect‐related processing. In the present study, gray matter changes in the right dorsal caudate showed significant directed associations with prefrontal regions, consistent with the idea that affective resonance may precede higher‐order cognitive evaluation during empathy. Early caudate responses may encode emotional valence via dopaminergic input from the ventral tegmental area, which in turn modulates prefrontal regulation.

The IFG, a critical node in the mirror neuron system, is implicated in action understanding and embodied emotion processing [39, 40]. Prior studies have associated IFG volume with emotion recognition abilities [41], whereas structural reductions in schizophrenia are linked to emotional blunting and social withdrawal [42]. Importantly, training‐based interventions such as meditation have been shown to increase IFG volume and enhance empathy [43]. In our network, the left IFG exhibited prominent directed connections, consistent with its integrative role in linking perceptual input from temporal regions with higher‐order representational processes.

Similarly, the STG, another component of the mirror neuron system, plays a central role in processing audiovisual information and biological motion. Structural enlargement of the STG has been reported in musicians and relates to prosodic processing [44], while developmental studies suggest coordinated maturation between the STG and medial prefrontal regions [45]. In the present analysis, bilateral STG functioned as structurally influential nodes of directed structural associations, underscoring their importance in organizing auditory–social information within the broader network.

In contrast, several regions were identified as targets of directed structural associations, including the bilateral SFG, right orbital gyrus, and bilateral postcentral gyri. The SFG contributes to multiple components of social cognition, with dorsal subdivisions supporting perspective‐related processing and ventral regions involved in emotion regulation and self–other distinction [46, 47]. Evidence that SFG volume increases with working memory training and correlates with cognitive empathy aligns with our finding that it functions as a recipient of structural influence, potentially supporting late‐stage regulation and perspective‐taking processes.

The orbital gyrus (orbitofrontal cortex [OFC]) encodes the social value of rewards [48] and modulates prosocial behavior [49]. Through its connections with the amygdala and insula, the OFC supports affective empathy [50]. Previous work has also shown that OFC morphology covaries with auditory cortical development, suggesting auditory–emotional integration [51]. Its high degree of incoming and outgoing directed connections in the present network supports a role as a structural hub coordinating information across multiple regions.

Finally, the postcentral gyrus, corresponding to primary somatosensory cortex, processes proprioceptive input and interacts with motor and parietal regions during sensorimotor integration [52, 53]. In our analysis, bilateral postcentral gyri predominantly appeared as target regions within the directed network, suggesting that their structural variability is systematically related to patterns in other cortical and subcortical areas. This organization may reflect fine‐tuning of embodied sensory representations within experience‐dependent SMNs.

### 4.2. Effects of Dance Training on Structural Similarity in the Empathy Network

Analysis of interregional gray matter distribution similarity revealed that dance training selectively modified structural coupling between the left STG and the left precuneus, left postcentral gyrus, and right paracentral lobule. As a core auditory node, the STG encodes pitch and frequency information, and prior work in ballet dancers has demonstrated increased STG volume following long‐term training [10]. The precuneus, a hub of motor imagery and self‐referential processing, integrates multimodal input during dance to synchronize movement and music [54]. This finding is consistent with their co‐activation during metaphorical and nonverbal emotional expression [55]. Importantly, this coupling was positively correlated with EC scores, suggesting that co‐plasticity between these regions may strengthen affective empathy. This interpretation aligns with autism studies reporting disrupted STG–precuneus connectivity in individuals with empathy deficits [56].

By contrast, STG–postcentral gyrus similarity was significantly reduced in dancers, potentially reflecting greater reliance on audiovisual–motor integration and reduced dependence on somatosensory feedback [57]. Interestingly, despite the absence of significant group differences in behavioral measures, this reduction remained positively correlated with FS scale scores, which index abstract emotional reasoning in fictional contexts. This apparent paradox may suggest that dance training may redistribute neural processing demands toward automated action–emotion coupling, thereby reducing the need for effortful inferential processing.

The paracentral lobule, an extension of the primary motor cortex, coordinates fine motor sequences. Enhanced STG–paracentral lobule similarity in dancers may reflect experience‐related coupling supporting movement–sound synchronization [58]. Notably, this coupling was positively associated with PD and negatively with PT. PD reflects self‐oriented affective arousal, whereas PT captures cognitive aspects of empathy. This dissociation suggests that stronger embodied emotional resonance may be associated with heightened affective reactivity, which does not necessarily translate into adaptive empathic understanding, as PD is often considered a maladaptive facet of empathy and may reflect increased emotional sensitivity [59]. Finally, increased structural similarity between the right paracentral lobule and right MTG was observed in dancers. The MTG is critical for interpreting action intentions [60], and this enhancement may reflect training‐induced reinforcement of action–affect mapping, supporting faster decoding of others’ motives.

Overall, these findings indicate that dance training is associated with alterations in structural similarity, characterized by altered coordination between auditory, motor, and socio‐emotional regions, which may reflect domain‐specific sensorimotor expertise related to action–emotion coupling.

## 5. Conclusion

This study applied GCA to construct a structural empathy network and identified its core nodes based on in‐degree and out‐degree measures. Findings suggest that source nodes of directed structural influence are primarily associated with the processes related to action observation–simulation and emotional sharing, while target regions contribute to adaptive expression of empathy via multimodal integration and cognitive regulation. We further examined the plasticity of this network in the context of dance training. Structural similarity analyses revealed that long‐term dance training is associated with enhanced cross‐modal integration between auditory, visual, and motor systems. This optimization appears to strengthen emotional mapping and increase affective resonance, while showing reduced structural coupling associated with cognitive empathy components. This pattern may reflect a potential trade‐off between emotional resonance and cognitive regulation during empathic processing. Taken together, these findings deepen our understanding of the structural foundations of empathy and highlight how domain‐specific expertise, such as dance training, can modulate the structural organization of neural networks underlying empathy.

## 6. Limitation

Several limitations of the present study should be acknowledged. First, while GCA enables inferences about directional dependencies among brain regions, its use with non‐temporal, structural data limits the strength of causal interpretations. Future work integrating longitudinal or task‐based functional imaging would enhance the temporal resolution and robustness of causal inference.

In addition, the limited sample size and the use of a a relatively homogeneous university student sample constrain the generalizability of the findings. Including more diverse expertise types and larger samples (e.g., athletes or non‐student professionals) would help clarify domain‐specific versus general effects. Finally, empathy was assessed using self‐report measures only, without objective behavioral tasks, limiting interpretation of the relevance of the observed structural network reorganization. Future studies combining structural and functional neuroimaging with behavioral measures of emotion recognition, PT, and prosocial decision‐making would help clarify the behavioral significance of experience‐dependent neuroplasticity.

## Funding

This work was supported by the National Key R&D Plan of China (Grant 2024YFE0215100), the National Natural Science Foundation of China (Grants 62401124, 62201133, and 82371560), the Natural Science Foundation of Sichuan Province (Grants 2023NSFSC0037 and 2022NSFSC0646), the China Postdoctoral Science Foundation (Grant 3480), the Chengdu Science and Technology Bureau (Grant 2024‐YF05‐02056‐SN), the CAMS Innovation Fund for Medical Sciences (CIFMS) (Grant 2019‐I2M‐5‐039), and the Sichuan Medical Association Scientific Research Project (Grant Q23045).

## Conflicts of Interest

The authors declare no conflicts of interest.

## Supporting Information

Additional supporting information can be found online in the Supporting Information section.

## Supporting information


**Supporting Information** Table S1. Permutation results for structural similarity between brain regions localized by Granger causal analysis. Figure S1. Null distribution for permutation results displayed.

## Data Availability

The datasets used and analyzed during the current study are available from the corresponding author upon reasonable request.
