# Supplementary material for: Exploring the Impact of Dance Training on the Structural Plasticity of Empathy‐Related Brain Networks
Source: Neural Plast. 2026 Apr 20;2026:2899142. doi: 10.1155/np/2899142 (PMC13094407; doi:10.1155/np/2899142)
Supplement: Supplementary file 1 — Supporting Information Table S1. Permutation results for structural similarity between brain regions localized by Granger causal analysis. Figure S1. Null distribution for permutation results displayed. [file NP-2026-2899142-s001.docx]

Supplementary Materials

Table S.1 summarizes the interregional connections showing significant KL divergence that remained robust after 10,000 iterations of permutation testing. These regions were identified as empathy-related structural nodes based on the GCA analysis. Furthermore, Figure S.1 illustrates the null distributions of F-values generated across the 10,000 permutations, with the empirical F-values overlaid, providing additional evidence for the robustness and reliability of the observed effects.

Table S.1 Permutation results for structural similarity between brain regions localised by Granger causal analysis

| **Brain regions** | **Abbr** | **Control** | **Dancer** | **Musician** | **F** | **P** |
| --- | --- | --- | --- | --- | --- | --- |
| Paracentral Lobule & Superior Temporal Gyrus | PCL_R_2_2& STG_L_6_4 | 0.83±0.12 | 0.89±0.07 | 0.85±0.12 | 3.53 | 0.01 |
| Paracentral Lobule & Middle Temporal Gyrus | PCL_R_2_2& MTG_R_4_1 | 0.80±0.12 | 0.85±0.08 | 0.84±0.08 | 2.98 | 0.03 |
| Precuneus & Superior Temporal Gyrus | PCun_L_4_3 & STG_L_6_4 | 0.70±0.15 | 0.78±0.12 | 0.74±0.16 | 4.06 | 0.002 |
| Postcentral Gyrus & Superior Temporal Gyrus | PoG_L_4_1 & STG_L_6_4 | 0.88±0.07 | 0.82±0.13 | 0.88±0.08 | 3.78 | 0.003 |


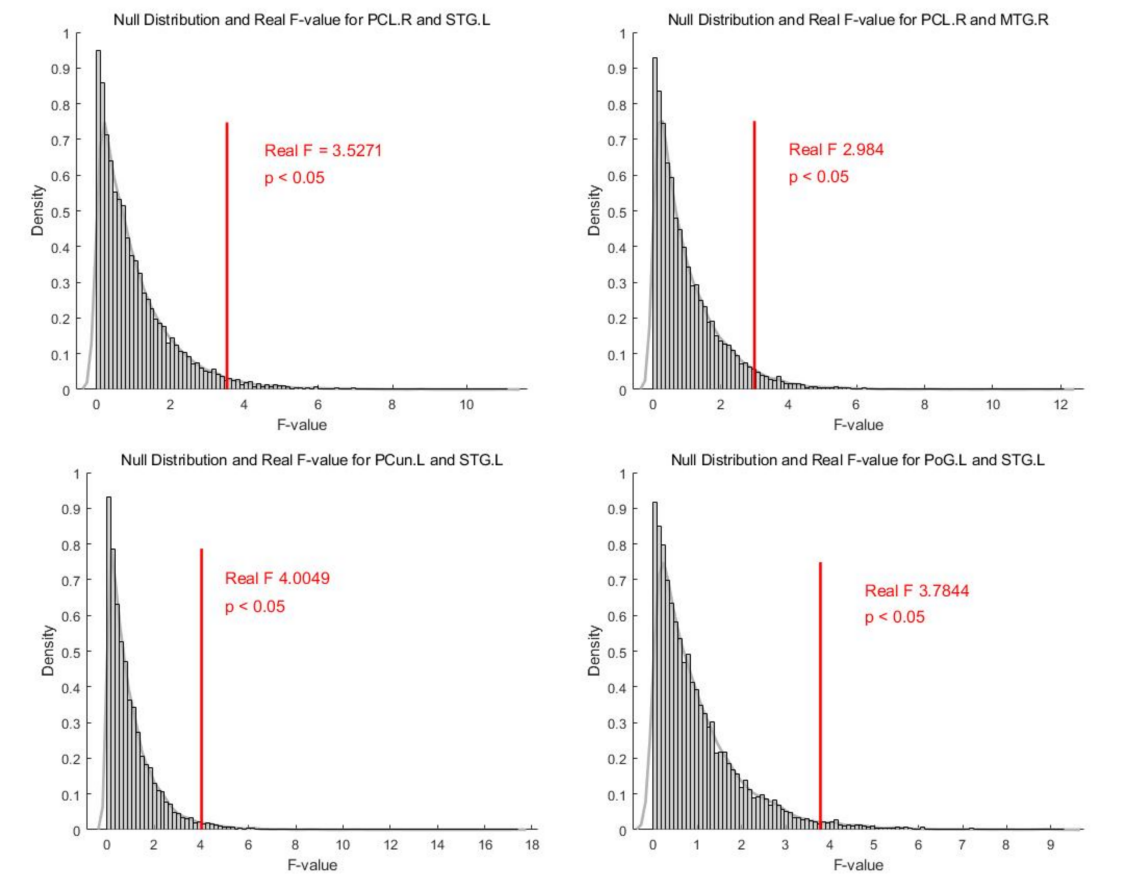


Figure S.1 Null distribution for Permutation results displayed in Table S.1
